# An anatomical and functional topography of human auditory cortical areas

**DOI:** 10.3389/fnins.2014.00225

**Published:** 2014-07-29

**Authors:** Michelle Moerel, Federico De Martino, Elia Formisano

**Affiliations:** ^1^Department of Cognitive Neuroscience, Faculty of Psychology and Neuroscience, Maastricht UniversityMaastricht, Netherlands; ^2^Maastricht Brain Imaging Center, Maastricht UniversityMaastricht, Netherlands; ^3^Department of Radiology, Center for Magnetic Resonance Research, University of MinnesotaMinneapolis, MN, USA

**Keywords:** human auditory cortex, tonotopy, ultra-high field fMRI, cytoarchitectonic parcellation, auditory cortical areas

## Abstract

While advances in magnetic resonance imaging (MRI) throughout the last decades have enabled the detailed anatomical and functional inspection of the human brain non-invasively, to date there is no consensus regarding the precise subdivision and topography of the areas forming the human auditory cortex. Here, we propose a topography of the human auditory areas based on insights on the anatomical and functional properties of human auditory areas as revealed by studies of cyto- and myelo-architecture and fMRI investigations at ultra-high magnetic field (7 Tesla). Importantly, we illustrate that—whereas a group-based approach to analyze functional (tonotopic) maps is appropriate to highlight the main tonotopic axis—the examination of tonotopic maps at single subject level is required to detail the topography of primary and non-primary areas that may be more variable across subjects. Furthermore, we show that considering multiple maps indicative of anatomical (i.e., myelination) as well as of functional properties (e.g., broadness of frequency tuning) is helpful in identifying auditory cortical areas in individual human brains. We propose and discuss a topography of areas that is consistent with old and recent anatomical *post-mortem* characterizations of the human auditory cortex and that may serve as a working model for neuroscience studies of auditory functions.

## Introduction: challenges for the investigation of the human auditory cortex

A major scientific approach in brain research has been to divide the cortex into smaller anatomical areas based on their micro-structural properties (Brodmann, 1909; Zilles and Amunts, 2009; Nieuwenhuys, 2012) and examine each area's functional properties through the analysis of the responses of neurons and neuronal populations. Whereas in animal models the link between micro-structural and functional properties of an area can be studied directly and in the same individual animal, in non-invasive research in humans such a link is much more labile, as it relies on the gross correspondence to macro-anatomical landmarks or matching to probabilistic atlases derived from post-mortem analysis of different brains (Morosan et al., 2001). Establishing an accurate parcellation of the cortical areas is thus essential in human research for studying the functional role of the various areas and for comparing results across experiments and laboratories. Furthermore, such a parcellation is crucial for understanding homologies and differences between human and animal cortex. Research into the visual system is a prominent example where such an approach has been successful. Functional magnetic resonance imaging (fMRI) has enabled mapping of the retinotopic organization in the human visual cortex *in vivo* and non-invasively (Engel et al., 1994; Sereno et al., 1995; Goebel et al., 1998). Because adjacent areas have opposite representations of the retinal image, the area borders can be outlined by calculating the sign of the local visual field (Sereno et al., 1995). With such an approach, the functional topography of early visual areas could be objectively mapped in individual human subjects and compared to topography of areas in the monkey visual cortex (Van Essen, 2004). This methodology provided a crucial tool for studying in detail the role of the distinct visual areas in visual information processing. Furthermore, similar methods have been used for discovering location and functional topography of high-order visual areas in both the ventral-temporal (Malach et al., 2002; Hasson et al., 2003) and parietal cortex (Sereno et al., 2001).

Despite the fact that fMRI research on the auditory system begun approximately at the same time as that on the visual system (see Talavage and Hall, 2012), to date there is no functional parcellation scheme of human auditory cortical areas that is generally accepted and routinely used across laboratories. While some of the impediments are of technical nature (e.g., the experimental limitations arising from the acoustic noise generated by the MR scanner, see Di Salle et al., 2003; Talavage and Hall, 2012), the main reasons remain exquisitely neuroscientific. First, there is no dominant model of anatomical parcellation of human auditory cortical areas. In the monkey, the auditory cortex presents a hierarchical organization with a core of primary auditory areas that receive ascending projections from the auditory portion of the thalamus, and is surrounded by non-primary belt and parabelt regions (Hackett et al., 1998, 2001). Each of these cortical partitions (i.e., core, belt, and parabelt) contains a number of auditory areas that can be distinguished based on their micro-anatomical and functional properties and their connectivity to sub-cortical structures and other cortical areas (Kaas and Hackett, 2000). This anatomical model of monkey auditory cortex is well-established and similar cortical models exist for a range of other species (Kaas, 2011). However, large differences exist between monkey and human auditory cortex even at macro-anatomical level. For example, in the human brain, the auditory cortex presents an expansion of cortical surface, with additional gyri and with a much larger inter-individual variability compared to the monkey (Galaburda et al., 1978; Hackett et al., 2001). Thus, when the goal is to define the detailed topography of auditory areas in individual human subjects, the monkey model may not be directly applicable. Studies of *post-mortem* anatomy indicate that the human auditory cortex contains a similar organization as in the monkey with core, belt, and parabelt subdivisions (Hackett et al., 1998; Morosan et al., 2001). But, strikingly, at the finer level of area definition, there are large differences among the various reports both with respect to the number of presumed auditory areas and to their location (see below).

Second, while in the visual system adjacent areas have opposite representations of the retinal image (Sereno et al., 1995), in the auditory system the frequency preference (i.e., the tonotopic gradient) is expected to run in parallel throughout the core and the directly adjacent belt area (Rauschecker and Tian, 2004). Thus, based on tonotopy maps alone, it is not possible to delineate precise areal borders. It is because of this intrinsic indeterminacy that—despite the feasibility of obtaining fMRI tonotopic maps of the human auditory cortex—a consensus regarding a tonotopy-based parcellation of the auditory areas has not yet been reached (Langers and van Dijk, 2012; Baumann et al., 2013; Saenz and Langers, 2014).

The aim of this review is to suggest a topography of the human auditory areas that may serve as a reference for fMRI studies of auditory functions. First, we review old and recent anatomical studies that provide a cyto- or myelo-architectonic characterization of the human auditory cortex with the goal of defining a consistent anatomical subdivision of the human auditory cortex and of reconciling reports that used different methods and different nomenclatures. Next, we show that the tonotopic maps found in different laboratories using different stimuli and acquisition/analysis methods are largely consistent. We demonstrate that whereas a group-based approach is appropriate to highlight the main high-low-high primary frequency gradient, the analysis of the maps at single subject level is required to detail the topography of areas and tonotopic gradients that may be more variable across subjects. Finally, we interpret the tonotopic maps in the light of recent characterizations of the human auditory cortex beyond frequency preference and propose a model that is compatible with both anatomical and functional characterizations of human auditory cortex.

## Anatomy of the human auditory cortex

### Macroanatomy of the human auditory cortex

**Figure 1:**
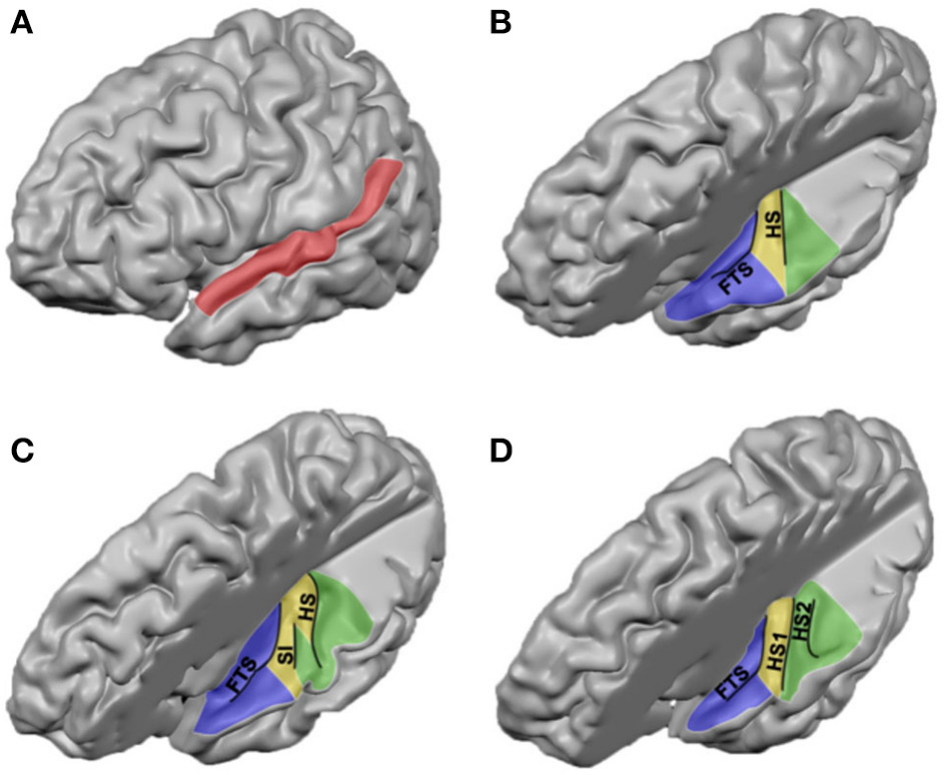
**Anatomical landmarks on the human supratemporal plane. (A)** Lateral view of the left hemisphere, with STG indicated in red. **(B–D)** Top view of left supratemporal plane, after removal of a large part of the parietal cortex. PP, HG, and PT are indicated in blue, yellow, and green, respectively. Major sulci are outlined in black (FTS, first transverse sulcus; SI, sulcus intermediate; HS, Heschl's sulcus; HS1, first Heschl's sulcus; HS2, second Heschl's sulcus). Panels include hemispheres with one HG, an incomplete separation of HG, and two HG in **(B–D)**, respectively.

The human auditory cortex is situated on the supratemporal plane, and comprises the superior two-thirds of the superior temporal gyrus (STG; Celesia, 1976; Galaburda and Sanides, 1980; Rivier and Clarke, 1997). On a macroscopic scale, the human auditory cortex can be divided in three regions (Kim et al., 2000; Figure 1). In anterior to posterior direction, the auditory cortex includes planum polare (PP), the transverse temporal gyrus or Heschl's gyrus (HG), and planum temporale (PT). HG is a convolution on the supratemporal plane, branching obliquely from the STG and hidden in the depth of the Sylvian fissure (SF). HG is evolutionary new: this convolution is not present in the macaque monkey (but see Baumann et al., 2013), and can be discerned in only a subset of chimpanzee brains (Hackett et al., 2001). There is considerable variability in the number of convolutions on the human supratemporal plane, ranging from one to three complete duplications of the transverse gyrus per hemisphere (compare Figures 1B–D; Campain and Minckler, 1976; Penhune et al., 1996). Besides complete duplications, a shallow intermediate sulcus (SI) may divide a single HG incompletely (Figure 1C). HG is bordered medially by the insular cortex, laterally by STG, and anteriorly and posteriorly by the first transverse sulcus and Heschl's sulcus, respectively (but see variations in Figures 1B–D). PT is posterior to HG. This triangular region is bordered medially by the SF, and laterally by the rim of the supratemporal plane. It shows a marked asymmetry and is consistently larger in the left hemisphere (Geschwind and Levitsky, 1968; Galaburda et al., 1978; Bonte et al., 2013). In humans, the PT region is much expanded compared to the monkey (Galaburda et al., 1978). Anterior to HG—separated by the FTS—lays PP, further delimited by the insula and the frontal operculum (Kim et al., 2000).

### Cytoarchitectonic subdivisions

**Figure 2:**
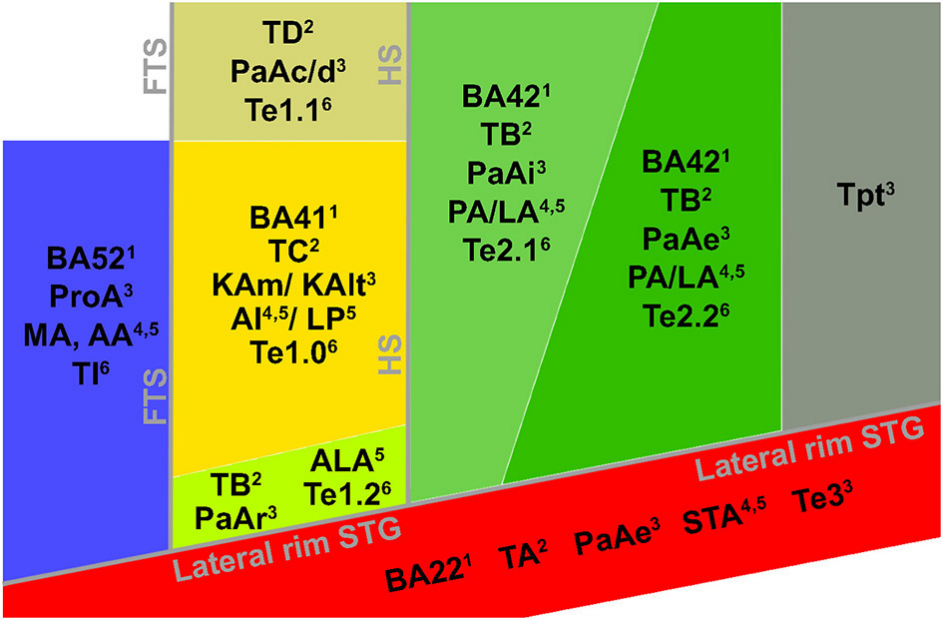
**Cytoarchitectonic characterization of human auditory cortex**. Major landmarks are outlined and named in gray. The layout and combinations are interpreted from Brodmann (1909)^1^, Von Economo and Horn (1930)^2^, Galaburda and Sanides (1980)^3^, Rivier and Clarke (1997)^4^, Wallace et al. (2002)^5^, Morosan et al. (2001, 2005)^6^, and Clarke and Morosan (2012)^6^.

In addition to describing the human auditory cortex in terms of its major anatomical landmarks, it has been labeled according to a variety of architectonic schema (Galaburda and Sanides, 1980; Rivier and Clarke, 1997; Hackett et al., 2001; Morosan et al., 2001). Across architectural studies, however, large differences exist with respect to the number of observed auditory areas, the location of these regions, and nomenclature. These differences already exist when parcellating HG, yet discrepancies between studies enlarge with increased distance from HG. Here, we present an overview of obtained results and propose how the different studies may be reconciled (see Table 1 and Figure 2).

**Table 1:** **Comparison of human cytoarchitectonics and primate fields**.

**Mapping study**	**PAC/core**	**Lateral belt**	**Parabelt**	**Medial junction**	**Medial belt**
Brodmann, 1909	41	42	42/22	41	52
Von Economo and Horn, 1930	TC	TB	TB/TA	TD	TG
Galaburda and Sanides, 1980	KAm, KAlt	PaAr, PaAi	PaAe	PaAc/d	ProA
Hackett et al., 2001—monkey cortex	A1, R	ML, AL	RP, CP	CM, CL	MM, RM
Rivier and Clarke, 1997; Wallace et al., 2002	A1, LP	PA, LA, ALA	PA, LA, STA		MA, AA
Morosan et al., 2001, 2005	Te1.0	Te1.2, Te2	Te2, Te3	Te1.1	TI
Myelin (Nieuwenhuys, 2012)	ttr1/ttrI	ttr2/ttrII	Lateral ts/tsep	ttr1/ttrI	Medial ts/tsep

Interpreted from Brodmann (1909); Von Economo and Horn (1930); Galaburda and Sanides (1980); Hackett et al. (2001); Morosan et al. (2001, 2005); Rivier and Clarke (1997), and Wallace et al. (2002). Regions defined by Hopf (1954) and Beck (1928) are included, as they were summarized in Nieuwenhuys (2012). The “medial junction” refers to the intersection of posteromedial HG, the retroinsula, and the medial aspect of the parietal operculum.

All cytoarchitectonic studies delineate homologs regions to monkey primary auditory cortex (PAC) or “core,” referring to the highly granular koniocortex within the auditory cortex (see yellow region in Figure 2). The core has a well-developed layer IV, presumably reflecting dense thalamic input from the auditory portion of the thalamus, the medial geniculate body (MGB). Layer III of the core can be characterized by the presence of small to medium sized pyramidal cells (Clarke and Morosan, 2012). Chemo-architectonically the core has a dense expression of AChE, cytochrome oxidase (CyOx), and parvalbumin in the neuropil of layer IV (Clarke and Morosan, 2012). Brodmann (1909) named the core auditory area BA 41 and it may correspond to area TC of Von Economo and Horn (1930). Rivier and Clarke (1997) confirmed the presence of a primary area using CyOx staining, and referred to it as AI. Morosan et al. (2001) refer to it as Te1. In accordance with the monkey auditory core, which includes two [auditory area 1 (AI), rostral field (R)] or three [AI, R, and rostrotemporal field (RT)] subdivisions (Rauschecker et al., 1995; Hackett et al., 2001), several studies divided the human core into subfields, most likely reflecting the human homologs of monkey AI and R (column “PAC/core” of Table 1; KAm and KAlt: Galaburda and Sanides, 1980; AI and LP: Wallace et al., 2002), and possibly RT (green field lateral to the core in Figure 2; PaAr: Galaburda and Sanides, 1980; Te1.2: Morosan et al., 2001; ALA; Wallace et al., 2002). Alternatively, the region lateral to the core could reflect an extension of the lateral belt (TB in Von Economo and Horn, 1930).

The position of the human PAC relative to sulcal and gyral landmarks is variable. While in the macaque monkey the core region is elongated along the rostro-caudal axis of the temporal lobe, in the chimpanzee—where a rudimentary HG appears in part of the brains—the core is roughly aligned to the main axis of HG that is oriented from posteromedial to anterolateral direction across the supratemporal plane (Hackett et al., 2001). In human brains, when only one HG is present, the core is confined to this HG and occupies its medial and central parts. However, when other combinations of HGs are present (occurring in the majority of the population), the PAC may extend postero-medially into medial HS and even onto the second HG (Galaburda and Sanides, 1980; Rivier and Clarke, 1997; Hackett et al., 2001; Morosan et al., 2001; Sweet et al., 2005). Importantly, the PAC has been reported to occupy approximately half of the HG volume (Rademacher et al., 2001). To date, a cytoarchitectonic analysis is needed to univocally determine the anatomical location of the PAC in humans (but see below for recent MR-based developments).

In monkey auditory cortex, a belt region is situated around the core. The belt contains various subdivisions, including the anterolateral field (AL), middle lateral field (ML), caudolateral field (CL), caudomedial field (CM), and middle medial field (MM) (Hackett et al., 1998, 2001; Rauschecker and Tian, 2004). Belt subfield CM seems to be intermediate in hierarchy between core and belt regions (Hackett et al., 2001). Immediately adjacent to the lateral belt on the exposed surface of the STG lay rostral and caudal parabelt (Hackett et al., 2001). In accordance with the monkey model, in the human cortex several less granular fields surround the PAC. The cell packing in these fields is less dense than in the core, and pyramidal cells in layer III are larger and more numerous (Clarke and Morosan, 2012). Occupying HS—posterior and immediately adjacent to the PAC—an area with a reduced granular structure compared to primary core areas (parakoniocortex) and with large pyramidal neurons in layer IIIc has been consistently reported (column “lateral belt” in Table 1; green regions in Figure 2; PaAi, Galaburda and Sanides, 1980; PA/LA, Rivier and Clarke, 1997 and Wallace et al., 2002; Te2, Morosan et al., 2001, 2005; region TB, Von Economo and Horn, 1930). Posterior to HS, bordering PaAi and extending along the STG, Galaburda and Sanides (1980) distinguished an additional region named PaAe (column “parabelt” of Table 1; posterior green region and red region in Figure 2). The medial part of this region may correspond to posterior BA 42, while its lateral part may correspond to BA 22 (Te3 in Morosan et al., 2005). At this approximate cortical region, other studies described subfields oriented in medial to lateral direction (areas PA, LA, and STA; Rivier and Clarke, 1997; Wallace et al., 2002; see Table 1 and Figure 2). Posterior to these regions extending toward the temporoparietal junction, area Tpt is located (gray region in Figure 2). Tpt extends beyond the PT, including the posterio-lateral STG, portions of the parietal operculum, and part of the supramarginal gyrus (Galaburda and Sanides, 1980; Sweet et al., 2005). Cytoarchitectonically, this is a transitional region between specialized sensory and more general cortex.

At the intersection of the most postero-medial end of HG, around the retroinsular region and the medial aspect of the parietal operculum lies a region described as parakoniocortex (PaAc/d) by Galaburda and Sanides (1980; column “posteromedial HG” in Table 1). While this region shares parakoniocortical features, its layer III pyramidal cells are smaller than in the lateral belt posterior and lateral to PAC (PaAi). This region may correspond to the most medial part of Te1 (Te1.1; Morosan et al., 2001), area TD of Von Economo and Horn (1930), and reflect the human homolog of monkey belt region CM (and possibly CL), the region intermediate in hierarchy between core and belt (Hackett et al., 2001).

Anteromedial to the PAC, at the border between insular and temporal cortex, another region is discriminated across studies (“medial belt” in Table 1). Galaburda and Sanides (1980) distinguish area ProA, which may roughly correspond to area AA or MA in Wallace et al. (2002), and BA 52 (Brodmann, 1909; blue regions in Figure 2). This area is characterized by its relatively thin cortical ribbon and prominent infragranular layers. It may reflect the human homolog of monkey medial belt areas. The description of correspondences between monkey and human auditory cortex beyond the PAC is complicated by evolutionary recent expanses in the human cortex (Galaburda et al., 1978), and the lack of a thorough establishment of functional properties of these auditory cortical areas in either monkey or human (Schreiner and Winer, 2007). Comparative studies are needed to further our understanding of homologies and differences in the functional neuroanatomy of the human and monkey auditory cortex. To this end, large progress is expected from the acquisition of functional MRI (fMRI) measurements in the monkey brain (Petkov et al., 2006; Remedios et al., 2009; Joly et al., 2011).

### Myeloarchitectonic parcellations of the supratemporal plane

In addition to parcellating the supratemporal plane based on its cell types and density (cytoarchitecture) or chemical pattern (chemoarchitecture), variations in myelin content provide for another possible subdivision (myeloarchitecture). In the macaque auditory cortex, myeloarchitectonic studies revealed that the auditory core can be discriminated from surrounding belt cortex by its heavy myelination, reflecting its high density of thalamocortical connections. Within the core region, the most caudally located A1 stains more heavily for myelin than R and RT (Hackett et al., 1998). After establishment of myeloarchitecture in the school of Vogt and Vogt (Nieuwenhuys, 2012), two influential parcellations of the human temporal lobe have been carried out by Hopf (1954) and Beck (1928). Beck divided the temporal lobe into six regions, while Hopf distinguished seven regions. Each of these regions could be distinguished in subregions that could then be further divided into areas, resulting in an impressive number of regions in both parcellation schemes (74 and 60 areas for Beck and Hopf, respectively).

Nieuwenhuys (2012) recently summarized these myeloarchitectonic schemes. As in the monkey, a densely myelinated region was defined on HG (ttr or ttr1; Nieuwenhuys, 2012), presumably reflecting human PAC. Myelin density was highest on the crown of HG, and decreased when moving from medial to lateral HG. While the caudal part of HG was astriate, with no stripe visible in layers IV/Vb due to a uniformly dense myelination, weaker myelination of layer Va in the rostral part of HG resulted in a unistriate pattern (layer IV was visible). This region of densest myelination could be divided further in medial-lateral direction (ttrIi and ttrIe in Beck, 1928) possibly reflecting the human homologs of monkey core fields A1 and R, and caudo-lateral direction (Hopf, 1954), possibly reflecting regions KAm/KAlt described in Galaburda and Sanides (1980).

While a correspondence between myeloarchitectonic and cytoarchitectonic schemes beyond the PAC remains highly tentative, it is consistently reported that myelination decreases with distance from HG most likely reflecting belt and parabelt regions. More specifically, posterior to HG on PT a bi-striate myelination has been reported (Hackett et al., 2001), resulting from the lower myelination of layers Va and VIa and resulting visibility of the inner and out stripes of Baillarger (layers Vb and IV, respectively). This region, most likely reflecting human lateral belt corresponds to region ttr2 in Hopf (1954; ttrII in Beck, 1928). Hopf (1954) further segregates ttr2 along the anterior/posterior axis, which resembles the distinction between Te2.1/Te2.2 (Clarke and Morosan, 2012) and PaAi/PaAe (Galaburda and Sanides, 1980). However, while ttr2 and Te2 occupy only PT, PaAe extends onto the STG. Here, both the parcellation by Morosan et al. (2005) and the myeloarchitectonic schemes of both Beck and Hopf (as described in Nieuwenhuys, 2012) discriminate a tertiary or parabelt type cortex (Te3 and the lateral part of ts/tsep, respectively, possibly including tpartr of Hopf). The medial part of ts/tsep is situated anterior to the densely myelinated core, and as such may correspond to regions ProA (Galaburda and Sanides, 1980), AA/MA (Rivier and Clarke, 1997; Wallace et al., 2002), BA 52 (Brodmann, 1909), and the monkey medial belt (Hackett et al., 2001).

While the cyto-, myelo-, and chemoarchitectonic parcellations each give different schemes and seem hard to reconcile at first glance, several studies emphasize that greater precision of boundary definition is achieved when multiple architectonic techniques are applied simultaneously (Hackett et al., 2001). A similar idea is pursued by Zilles et al. (2002), which mapped the human cortex based on multiple transmitter receptors (Zilles et al., 2002; Morosan et al., 2005). They found that human PAC contained a high density of cholinergic muscarinic M2 and nicotinic receptions, most densely expressed in middle cortical layers. Both M2 and nicotinic receptor density sharply dropped at the lateral border of PAC with the belt (Clarke and Morosan, 2012). The combination of cyto-, myelo, and receptor architecture mapping (by staining alternating brain slices with different methods) applied to regions beyond the auditory core may in the future provide the unification of the studies summarized above.

## Tonotopic maps in the auditory cortex

### Tonotopy in the non-human primate

Numerous studies have investigated tonotopy—the orderly spatial representation of a neuron's preferred sound frequency—in the auditory cortex. Although tonotopy has been shown to break down at the level of individual cortical neurons (Bandyopadhyay et al., 2010; Rothschild et al., 2010), at a larger spatial scale tonotopic maps can reliably be found in the auditory cortex across species (Merzenich et al., 1973; Reale and Imig, 1980; Morel et al., 1993; Bendor and Wang, 2005). In primates, tonotopic maps are present in the core auditory region (Merzenich and Brugge, 1973), with reversals in the frequency gradient indicating the borders between the separate auditory fields (AI, R, and RT). The low-frequency border shared between AI and R and high-frequency border between R and RT appear to coincide with histologically defined borders (Merzenich and Brugge, 1973; Morel et al., 1993; Kaas and Hackett, 2000). The frequency selectivity or sharpness of tuning—reflecting the range of frequencies to which a neuron responds—is narrowest in core regions (Rauschecker et al., 1995; Hackett et al., 1998; Rauschecker and Tian, 2004; Kajikawa et al., 2005; Kusmierek and Rauschecker, 2009). In the belt areas, a number of auditory fields (e.g., AL, ML, CL, CM, and MM) have also been shown to contain a tonotopic map (Merzenich and Brugge, 1973; Rauschecker et al., 1995; Kosaki et al., 1997; Rauschecker and Tian, 2004; Kusmierek and Rauschecker, 2009). The primary frequency gradient (in the regions R, AI, and CM) runs parallel to the gradient in belt areas (AL, ML, and CL, respectively; Rauschecker and Tian, 2004). Consequently, reversals in the tonotopic gradient, used to divide core and belt into subfields, cannot be used to distinguish core from belt auditory cortex. Tuning width of neurons is commonly used to achieve this feat, as neurons in belt regions have a broader tuning width than those in core areas (Rauschecker et al., 1995; Hackett et al., 1998; Rauschecker and Tian, 2004; Kajikawa et al., 2005; Kusmierek and Rauschecker, 2009). Auditory cortex beyond the belt is not well-characterized in terms of its tuning to acoustic features (e.g., frequency preference, selectivity, and spectral/temporal modulations; Schreiner and Winer, 2007).

### Tonotopic maps in the human auditory cortex

FMRI studies in humans have partially confirmed the functional organization of the monkey auditory system. Early studies (Bilecen et al., 1998; Talavage et al., 2000; Engelien et al., 2002; Schönwiesner et al., 2002) gathered evidence for the presence of multiple frequency-selective responses along the Heschl's region, but failed to obtain detailed topographical representations of these frequency-selective responses. In one of the first neuroscientific applications of ultra-high field MR (7 Tesla), Formisano et al. (2003) depicted the detailed tonotopic layout of human PAC. Based on the spatial arrangement and mirror-symmetry of the frequency-selective responses, this tonotopic map was interpreted as reflecting the human homologs of monkey areas A1 and R (hA1 and hR; Merzenich and Brugge, 1973; Merzenich et al., 1973; Reale and Imig, 1980; Kaas and Hackett, 2000).

**Figure 3:**
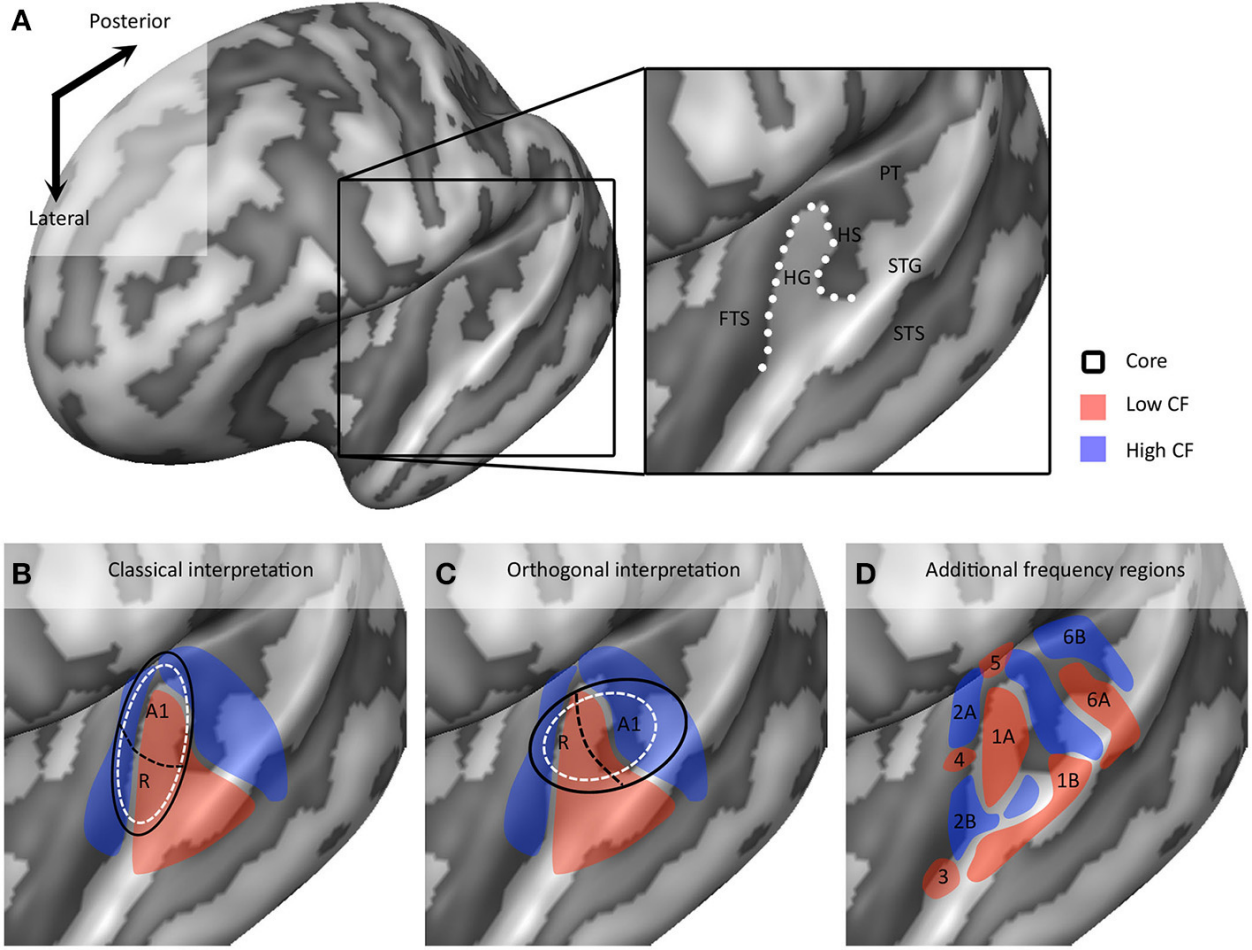
**Interpretations of tonotopic maps. (A)** Inflated representation of the left hemisphere. Light and dark colors reflect gyri and sulci, respectively. The black square outlines the part of cortex highlighted in the rest of the figure. The macro-anatomy of the auditory cortex is displayed on the right, showing Heschl's gyrus (HG), first transverse sulcus (FTS), Heschl's sulcus (HS), planum temporale (PT), superior temporal gyrus (STG), and superior temporal sulcus (STS). The white dotted line outlines HG. **(B,C)** Classical and orthogonal interpretation of tonotopic maps respectively, with core regions A1 and R outlined in black. Dashed white circles indicate variations on the models that cannot be excluded based on tonotopic maps alone. **(D)** Additional frequency selective gradients. In **(B–D)** red and blue colors show regions of low and high frequency preference, respectively.

In recent years, the extraction of tonotopic maps throughout the human superior temporal plane with fMRI has become increasingly feasible (Talavage et al., 2004; Woods et al., 2009, 2010; Humphries et al., 2010; Da Costa et al., 2011; Striem-Amit et al., 2011; Langers and van Dijk, 2012). Resulting maps show good correspondence across studies. A large low frequency region on HG is consistently observed, surrounded posteriorly (on HS and PT), antero-medially, and antero-laterally (on PP) by regions preferring high frequencies (Figures 3B,C). The regions preferring high frequencies adjoin at the medial end of HG, creating a “V” shaped pattern (blue regions in Figures 3B,C). It is commonly agreed that the human auditory core is situated within this main high-low-high gradient, yet studies vary widely in the exact part of the tonotopic gradient that they assign to the core (Baumann et al., 2013; Saenz and Langers, 2014). Interpretations vary from a placement of the auditory core along HG (“classical interpretation,” Figure 3B), to a placement across HG (“orthogonal interpretation,” Figure 3C) and everything in between (Baumann et al., 2013). The classical interpretation is in agreement with cytoarchitectonic investigations of human auditory cortex that reliably place the core on the medial and central part of HG. However, as the long axis of monkey auditory core runs parallel to the STG, across-species consistency may favor a perpendicular (Da Costa et al., 2011) or oblique (Baumann et al., 2013) arrangement of the core. Moreover, while some studies interpret the complete high-low-high map, stretching from PP to PT, as reflecting two primary auditory fields hA1 and hR (Da Costa et al., 2011), other studies suggest that part of this large gradient reflects auditory belt fields (Talavage et al., 2004; Woods et al., 2009, 2010; Humphries et al., 2010; Striem-Amit et al., 2011). Beyond the main high-low-high frequency gradient, an additional low frequency region is often reported at the antero-lateral border of the main gradient on PP/anterior STS (region 3 in Figure 3D; Talavage et al., 2004; Woods et al., 2009; Humphries et al., 2010; Moerel et al., 2012). Together with part of the anterior high frequency part of the main gradient, this region may reflect the human homolog of primary region RT (hRT).

### Additional frequency gradients in fine-grained tonotopic maps

**Figure 4:**
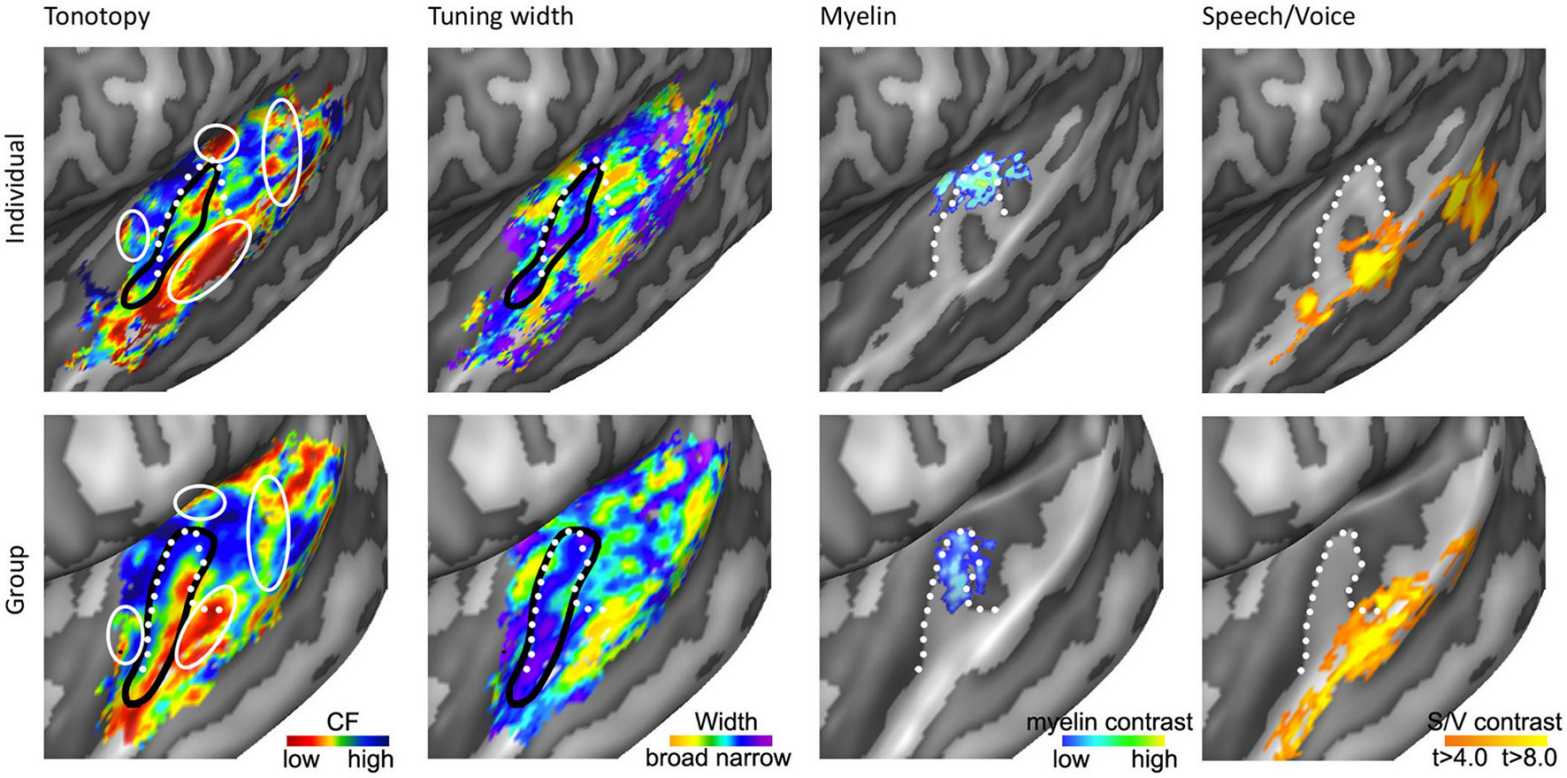
**Anatomical and functional characterization of human auditory cortex**. The four columns show tonotopy, tuning width, myelin contrast, and speech/voice selectivity in the left hemisphere of a single subject (**top**) and as group results (**bottom**; adapted from Moerel et al., 2013; De Martino et al., 2014). The black lines in the first and second column outline PAC, and the white circles in the first column indicate frequency regions beyond the main high-low-high tonotopic gradient. The white dotted lines show the location of HG.

As we explore tonotopic maps at higher spatial resolution, refrain from smoothing maps with large spatial filters, and inspect single subject maps, it becomes apparent that the auditory cortex contains a larger number of frequency reversals than commonly assumed (see Figure 3D). These additional gradients on the supratemporal plane are evident in individual subject maps, yet possibly due to their small extension and relatively variable location across individuals, are often not evident on group maps. Consequently, they are generally not discussed. Although we acknowledge that care must be taken with over-interpreting small regions, there are four patterns beyond the main gradient that consistently appear in single subject tonotopic maps (indicated with white circles in the tonotopic maps of Figure 4). These patterns may provide important information for defining a functional topography of human auditory areas.

First, the large low frequency region on HG and adjacent STG can be divided into two smaller regions (indicated with numbers 1A and 1B in Figure 3D; see regions “4” and “6” in left and right hemisphere, respectively in Striem-Amit et al., 2011, and the progression between endpoints 3′ and 6′ in Talavage et al., 2004). An ellipse-shaped low frequency region of which the long axis runs along HG is located in the middle of HG; this part of the large low frequency region most likely belongs to the auditory core (region 1A in Figure 3D; included within the black outlines on the tonotopic maps in Figure 4). A larger low frequency region can be discriminated on lateral HG/middle STG (region 1B in Figure 3D; white circle on middle STG in Figure 4). While these two low frequency regions often merge into one large frequency patch, only the medial region on HG belongs to the high-low-high core gradient. The region on lateral HG/STG may be part of the lateral belt.

Second, the large high frequency region on the anterior part of the auditory cortex (regions 2A and 2B in Figure 3D) is divided into two smaller regions by a small low frequency region (region 4 in Figure 3D, and most anterior white circle in Figure 4). This small low frequency region appears in a substantial number of single subject maps across fMRI investigations (Da Costa et al., 2011; Herdener et al., 2013; Moerel et al., 2013; see Figure 4), and may reflect fields of the human medial belt cortex (Brodmann area 52; Brodmann, 1909).

Third, another reversal in frequency is present on the posteromedial end of HG (region 5 in Figure 3D and medial white circle in Figure 4; Talavage et al., 2004; Da Costa et al., 2011; Langers, 2014). This region may correspond to monkey regions CM/CL, which have been reported to contain tonotopic gradients and share a high frequency border with A1 and ML, respectively (Kajikawa et al., 2005).

Finally, posterior to the main high-low-high frequency gradient, extending from HS covering PT and posterior STG, additional frequency regions are located (regions 6A,B in Figure 3D). A low frequency region has been reported (Humphries et al., 2010; Da Costa et al., 2011 describe this reversal attributing it mainly to the right hemisphere; Langers, 2014; the frequency progression between endpoints 3′ and 8′ in Talavage et al., 2004), and in many maps additional clusters preferring high frequencies are additionally present (posterior white circle on PT in Figure 4). Depending on the orientation of the auditory core, these regions have been interpreted as reflecting lateral belt and parabelt cortex (classical interpretation; Barton et al., 2012; Moerel et al., 2012), or CM/CL (orthogonal and oblique orientations; Humphries et al., 2010; Striem-Amit et al., 2011; Baumann et al., 2013). Because of large differences in anatomy between human and non-human PT (Hackett et al., 2001) and a relatively poor functional characterization of the monkey cortex beyond belt (Schreiner and Winer, 2007), we can no longer build on results from the non-human primate for an interpretation and parcellation of this region of cortex. Based on human cytoarchitecture studies, this region should include PaAe (Galaburda and Sanides, 1980; Te3, Morosan et al., 2005; LA/PA/STA, Wallace et al., 2002) and Tpt (Galaburda and Sanides, 1980). Understanding the precise topology of these regions requires additional knowledge regarding their response properties (e.g., tuning to temporal/spectral modulations, latency).

### Magneto-encephalographic and electrophysiological recordings of frequency selective auditory cortical responses

Major contributions to our current knowledge of the functional topography of human auditory cortex come from methods other than fMRI. Using MEG, the presence of a tonotopic organization has been investigated by exploring frequency-dependent shifts of auditory-evoked responses (AEFs). An early MEG study showed that evoked responses increased in depth (i.e., toward medial HG) with increases in frequency, presumably reflecting the tonotopic gradient in hA1. The observed tonotopic progression was described as a logarithmic mapping, in which the evoked response displaced as a function of the logarithm of the stimulus' frequency (Romani et al., 1982). MEG investigations of the human tonotopic organization since this study present conflicting outcomes. While some studies did not observe evidence of a tonotopic organization in their data (Roberts and Poeppel, 1996), the majority of MEG studies report postero-medial shifts of the equivalent dipole location with increasing frequency in agreement with Romani et al. (1982; N19m-P30m response in Scherg et al., 1989; N100m in Pantev et al., 1988; steady state response in Wienbruch et al., 2006). Findings of invasive (intracranial) electrophysiological recordings in humans are in accordance with this pattern. They observed that the neuron's CF increased toward postero-medial locations, supporting the presence of one tonotopic gradient on human medial HG (Howard et al., 1996). However, using MEG several other tonotopic patterns were observed as well, including one frequency gradient with reversed direction (compatible with the low-to-high gradient in hR; Hari and Mäkelä, 1986) and a mirror symmetric pattern (Pantev et al., 1995; N100m and Pam response reflecting high-to-low and low-to-high pattern when moving from postero-medial to antero-lateral locations, respectively). Throughout MEG studies, reported gradients are reproducible within an individual but highly variable across individuals (Lütkenhöner et al., 2003).

The variability across individuals and studies may be explained when considering advantages and disadvantages of using MEG as a tool for tonotopic mapping. While MEG does not suffer from fMRI drawbacks such as relatively low temporal resolution or interference of scanner noise, MEG is limited by other factors when mapping tonotopy (see discussion in Formisano et al., 2003). First, equivalent dipole modeling of neural activity originating from simultaneously active locations is problematic, and resulting dipoles generally reflect the combined activity from these sources. Tonotopic gradients within human auditory cortex are close to each other in time and space, and may therefore not be distinguished from each other using MEG. Second, because the multiple tonotopic gradients in subdivisions of human auditory cortex are variously oriented, it is not possible to distinguish whether an observed shift of dipole location with frequency originates from one tonotopic gradient, or from the relative weighting of these subdivisions. Again, this will have the result that MEG may not be able to distinguish frequency gradients correctly from each other. Third, interpretation of the N100m component that most MEG tonotopy studies are based on is controversial. While some studies interpret it as reflecting activity in PAC, substantial evidence points to it originating from secondary auditory areas such as PT instead (Lütkenhöner and Steinsträter, 1998; Engelien et al., 2000). This is supported by investigations of the anatomical origin of auditory evoked potentials using invasive electrophysiological recordings, which ascribed the generator of the N100 to PT and possibly the lateral part of HG (Liégeois-Chauvel et al., 1994). Thus, while MEG is well-suited to capture the dynamics of auditory processing, and has made substantial contributions to for example the investigation of cortical speech processing (Lütkenhöner and Poeppel, 2009), it is not optimal for mapping the relatively small frequency gradients within the human cortical tonotopic map.

Alternatively, invasive (intracranial) electrophysiological recordings (Liégeois-Chauvel et al., 1994; Howard et al., 1996; Nourski et al., 2014) have good spatial and temporal resolution, and thereby provide a unique window into the workings of human auditory cortex. For example, using invasive electrocorticography (ECoG) a recent study observed a dynamic mirror-symmetric tonotopic gradient on postero-lateral STG, supporting that cortical tonotopy maps extend far beyond the auditory core (Striem-Amit et al., 2011; Moerel et al., 2012). However, invasive electrophysiological recordings have limited applicability (i.e., mostly restricted to patients undergoing neurosurgical procedures for epilepsy or brain tumor) and spatial coverage (i.e., grid placement is determined by clinical criteria). As such, these measurements have not yet been able to provide a complete picture of spectral selectivity throughout the supratemporal plane.

## Characterizations of auditory cortex beyond tonotopy

### Limitations of tonotopic maps

Based on results from the monkey auditory cortex, the frequency gradient in the human core is commonly assumed to run parallel to the gradient in belt areas (Rauschecker and Tian, 2004). Consequently, the auditory cortex cannot be divided into core, belt, and parabelt based on maps of tonotopy alone. This creates several omissions in our knowledge of the human auditory cortex. As frequently discussed in the auditory neuroscience community, tonotopic maps alone are insufficient to determine the *orientation* of the auditory core with respect to HG (classical, orthogonal, or oblique; compare maps in bottom row of Figure 3). Equally important is the impossibility to determine the *size* of the human core based on tonotopic maps. For example, in the bottom row of Figure 3 the auditory core can be equally well-represented by the black lines and the white dotted lines. Cytoarchitectonic parcellations of the auditory cortex showed that the average size of the human auditory core is approximately 1650 mm^3^, roughly half of the entire HG (average size of HG = 3200 mm^3^). The size of the auditory core, and the relation between the size of the core and the size of HG, was shown to vary greatly across individuals (Rademacher et al., 2001). These results should be taken into account when interpreting tonotopic maps. While it is commonly agreed that the auditory core must include HG, the several studies interpret not only the entire HG but also surrounding areas on PP and PT (Da Costa et al., 2011; Herdener et al., 2013; Langers, 2014), leading to a substantial overestimation of the auditory core size. Finally, macroanatomy, microanatomy, and tonotopic pattern vary substantially across individuals (Rademacher et al., 2001; Da Costa et al., 2011). Part of the tonotopic map varies with macroanatomy, such that the main low frequency patch is likely to move in a posterior direction in the case of partial or complete duplications of HG (Da Costa et al., 2011). However, with only tonotopy as a characterization of the auditory cortex, *interindividual variation* in cortical organization—including interindividual variation in the orientation of the core—cannot be resolved (Rademacher et al., 2001). Reliable estimates of core size and location in individuals are crucial if we are to systematically study the functional properties of auditory fields, the transformations of sound representations throughout these fields, and deviations in special cases (e.g., musicians, tinnitus, or cochlear implants patients).

### *In vivo* mapping of myelo-architecture

Recent studies have explored functional and anatomical properties of the auditory cortex beyond its frequency preference. One promising research stream is to map cortical myelin density non-invasively using MRI (Glasser and Van Essen, 2011; Dick et al., 2012). While exploring cortical myelin density can only be performed on post-mortem tissue, recent studies showed that MRI contrast can reveal myelin-related maps *in vivo*. Specifically, myelin-related maps have been created using either quantitative T1 (Sigalovsky et al., 2006; Dick et al., 2012; Sereno et al., 2012), quantitative T2^*^ (Cohen-Adad et al., 2012), or based on T2 or T2^*^ weighted contrasts (Glasser and Van Essen, 2011; De Martino et al., 2014). In agreement with post-mortem studies, experiments mapping myelin non-invasively in the human cortex with MRI revealed a heavily myelinated region on the superior temporal plane. Specifically, anterior regions (PP) showed the least myelination, posterior regions (PT) were moderately myelinated, and HG was found to be most densely myelinated (Sigalovsky et al., 2006; Glasser and Van Essen, 2011). The highly myelinated region on HG coincided with probabilistic cytoarchitectonic regions Te1.1 and Te1.0 (Morosan et al., 2005; Glasser and Van Essen, 2011), and with two mirror-symmetric tonotopic gradients oriented along HG that were interpreted as reflecting hA1 and hR (Dick et al., 2012). The medial part of this mirror-symmetric gradient showed a slightly greater myelination than the lateral part. De Martino et al. (2014) partially replicated these findings at 7T. Using a clustering approach and multiple MR-contrasts, they automatically identified the most densely myelinated region in individual hemispheres. This region overlapped with a single high-to-low frequency gradient (see third column in Figure 4), and was interpreted as reflecting hA1. Importantly, both studies showed that myelin-related contrast varied among hemispheres and individuals, illustrating the need to obtain a distinct measure beyond tonotopy in order to identify the core in individual hemispheres (Dick et al., 2012; De Martino et al., 2014).

### Functional cortical tuning beyond frequency

In addition to cortical myelin contrasts, functional properties may provide crucial information on the auditory cortical organization. In the monkey auditory cortex, cortical tuning width is employed to distinguish core from belt areas. Tuning width refers to the frequency selectivity of a neuron, which is narrower in core than in belt regions (Rauschecker et al., 1995; Hackett et al., 1998; Rauschecker and Tian, 2004; Kajikawa et al., 2005; Kusmierek and Rauschecker, 2009). Recent studies used a computational model to analyze responses to natural sounds measured with fMRI, and thereby obtained maps of tuning width throughout the human auditory cortex (Moerel et al., 2012; De Martino et al., 2013). Regions of both narrow and broader tuning could be identified throughout the supratemporal plane. A narrowly tuned region along HG was evident in both hemispheres (second column of Figure 4). When only interpreting the narrow part of the tonotopy map as the PAC, a high-low-high-low tonotopic gradient was distinguished running in antero-lateral direction along HG (Figure 4). This region was identified as reflecting hA1, hR, and hRT. Note that tuning width maps only reflect the width of the main spectral peak, and therefore do not convey information regarding the complexity of spectral tuning (Moerel et al., 2013). Furthermore, as each fMRI voxel combines the signal coming from a substantial cortical patch and a large number of neuronal populations, the tuning width maps may reflect at least in part the homogeneity of neuronal spectral tuning rather than the tuning width alone. Consequently, while tuning width maps may be used to identify PAC in individuals, they may not be informative for regions beyond HG.

As natural sounds can be characterized well by their energy modulations in the spectral and temporal dimensions, it has been suggested that preferential processing of these auditory features may crucial to describe the topography of the auditory cortex. Indeed, in the monkey auditory midbrain (Baumann et al., 2011) and cat auditory cortex (Langner et al., 2009), a map of periodicity preference orthogonal to the tonotopic map has been observed. Periodicity refers to the rate of temporal modulations in a sound, which evokes a corresponding pitch percept. Recent studies used fMRI to map frequency and periodicity preference throughout the human auditory cortex (Barton et al., 2012; Herdener et al., 2013). Based on the combination of these maps, Barton et al. (2012) parcellated the auditory cortex into “clover leaf” clusters (Barton et al., 2012). Within this parcellation scheme, tonotopic reversals serve to segregate cloverleaf clusters from each other, while periodotopic reversals divide a cluster into auditory fields. In this manner 11 auditory subfields were identified, with core regions hA1 and hR occupying medial and lateral HG, respectively. Conversely, Herdener et al. (2013) observed a gradient of periodotopic preference along HG, with medial and lateral parts preferring high and low temporal modulations, respectively. This discrepancy in results is so far not explained when simultaneously exploring preference to combined spectral and temporal modulations, either using artificial sounds (“ripples”; Langers et al., 2003; Schönwiesner and Zatorre, 2009) or complex natural sounds (Santoro et al., 2014). While the cortical spectral modulation preferences revealed by these studies consistently showed that regions along and antero-ventrally to HG process fine-grained spectral information, such consistency across studies was not apparent with regard to resulting maps of temporal modulation preference (i.e., periodotopy). Future studies will be needed to elucidate these findings.

Beyond large-scale maps of feature preference, research in primates (Petkov et al., 2008) and humans (Belin et al., 2000; Zatorre et al., 2002) indicates that the non-PAC contains regions where neuronal populations respond stronger to conspecific vocalizations than to other sound categories (i.e., speech and voice regions, see Figure 4). These speech/voice regions contribute to the formation of higher-level sound representations, at least partially abstracted from the sound acoustics (Belin et al., 2000; Formisano et al., 2008). A recent exploration of the relation between these higher level regions and low level feature maps revealed a consistent overlay of speech/voice regions and the low frequency part of tonotopic maps (Moerel et al., 2012; compare first and last columns of Figure 4). This overlap was present even when simple tones were presented and was interpreted as reflecting a specialized filter mechanism, enhancing those low level features (i.e., the low frequencies) crucial to speech and voice sounds. These results suggest that—similar to eccentricity mapping in the visual system (Malach et al., 2002)—tonotopic mapping may also help defining the topography of high-order auditory areas.

## A working model of human auditory cortex

### The orientation and size of the human core

**Figure 5:**
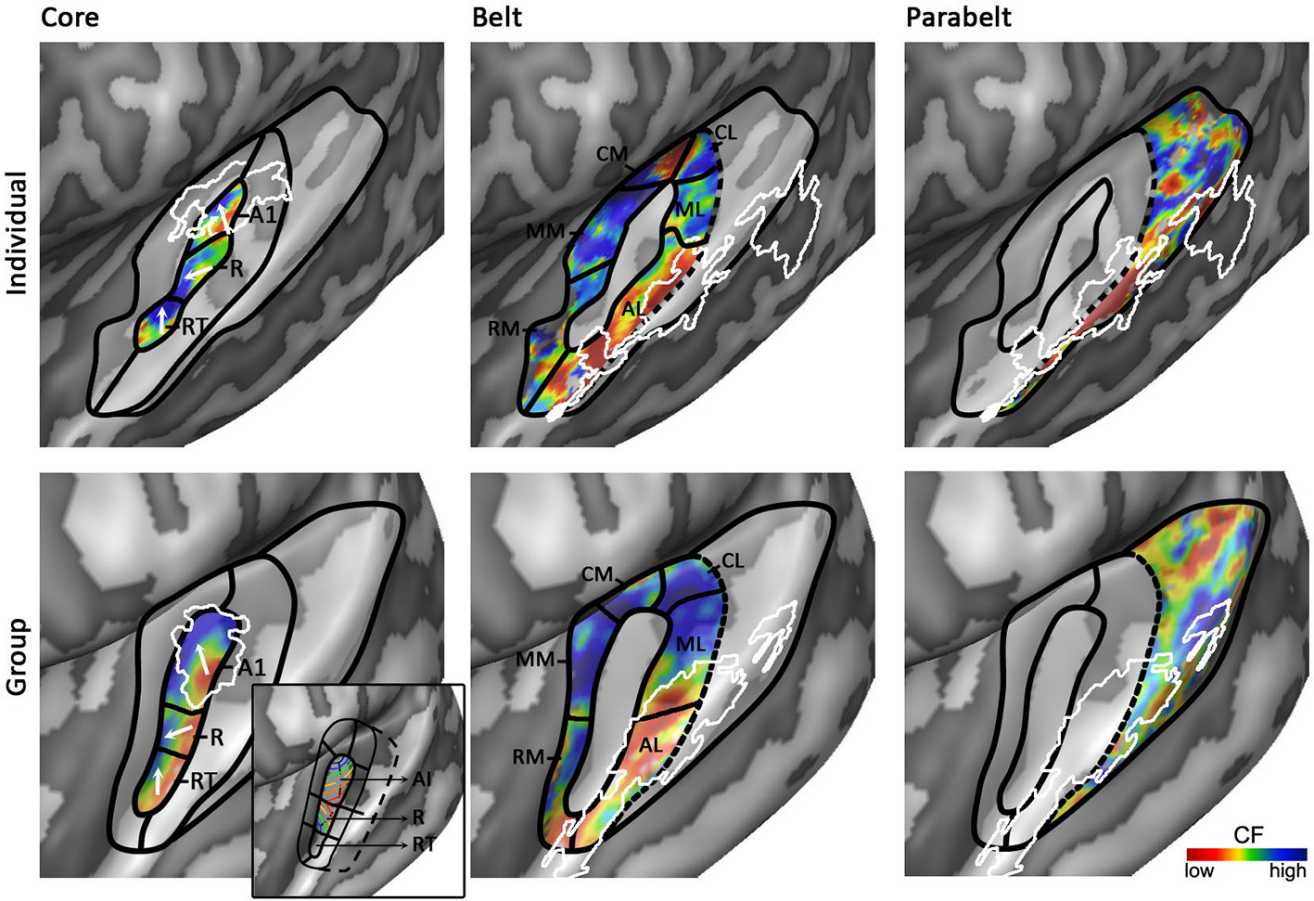
**Working model of human auditory cortex**. Parcellation of the auditory cortex into core (left), medial and lateral belt (middle), and parabelt (right) based on individual (**top**) and group (**bottom**) data. Solid black lines indicate boundaries between auditory fields based on maps of tonotopy, tuning width, or myelin. Dotted black lines indicate boundaries assumed based on literature, but for which no objective measure is available. White outlines indicate maps of myelin (left) or speech/voice selectivity (middle and right). The white arrows in the first column show the main direction of the frequency gradient within the three core fields. The inset in the panel “Group – Core” shows the primate model of auditory cortex as interpreted and adapted from Kaas and Hackett (2000). The model is rotated so that the core aligns with HG. Isofrequency gradients are color-coded to match the colorscale in our tonotopy maps.

Over a decade after the first fMRI studies showing tonotopic maps in the human auditory cortex, the discussion of how these maps should be interpreted is still at full force (Langers and van Dijk, 2012; Baumann et al., 2013; Saenz and Langers, 2014). To add to this discussion, we propose a working model of the human auditory cortex (Figure 5) and attempt to reconcile results from high-resolution tonotopic mapping and other non-invasive functional characterizations with results from *post-mortem* and *in vivo* anatomical studies. Furthermore, we discuss similarities and divergence with respect to the commonly accepted model of monkey auditory cortex (Hackett et al., 1998).

Based on human cytoarchitectonics, the core size should on average correspond to half of HG. Furthermore, it should be largely restricted to HG, yet deviations from HG can occur at the postero-medial end especially in the case of partial or complete duplications. In individual subjects the core should coincide with a narrowly tuned region of tuning width maps. Accordingly, we place the core largely in the medio-lateral direction of HG, oriented at a relatively small angle from the long direction of the STG (Figures 4, 5). The medial part of the core coincides with the region of highest myelination. This orientation of the core is compatible with the macaque model. In a recent review, Baumann et al. (2013) clarified that contrary to common assumption the macaque auditory cortex contains a protuberance that may be interpreted as the precursor of human HG. The main high-low-high tonotopic gradient runs at a slight angle with respect to this protuberance, extending slightly beyond its postero-medial and antero-lateral endpoints. The proposed core region in Figures 4, 5 is consistent with this arrangement. Note that while our proposed region is very similar in orientation compared to the region proposed by Baumann et al. (2013), there is a crucial difference in the location of hA1. Baumann et al. (2013) proposed to place hA1 in the medial HG with overlap into medial and central HS and possibly extending onto PT. Instead, in our model hA1 occupies medial locations including medial HG and medial HS, but excludes the most lateral part of hA1 (i.e., the extension of hA1 into central HS/PT) as proposed by Baumann et al. (2013).

The first high frequency maximum occupies the most medial part of HG. The main high-to-low frequency gradient, reflecting hA1, proceeds laterally ending in the main low frequency maximum. The tonotopic gradient reverses direction at this low frequency maximum and travels to the second high frequency maximum on anterior and lateral HG. This creates the second complete frequency map, reflecting hR. The frequency gradients in hA1 and hR run at an approximately 90° angle to each other (see two white arrows in hA1 and hR in Figure 5). The organization of the resulting human PAC tonotopy model is strikingly similar to what was proposed for the non-human primate. Compared to the model as proposed by Hackett et al. (1998, 2001; see Kaas and Hackett (2000) for the orientation of isofrequency bands within the core), the current PAC is rotated to align with HG but its tonotopic organization remains identical (compare human tonotopy gradient in Figure 5 “Group – Core” to primate model inset in this panel). A second frequency gradient reversal occurs in antero-lateral HG (reflecting hRT; most anterior and lateral white arrow in Figure 5), resulting in a high-low-high-low tonotopic pattern within human PAC.

### Exploring belt and parabelt regions

An auditory responsive region surrounds the core, possibly reflecting the medial and lateral belt (middle part of Figure 5). In the medial portion, a small low frequency region divides the large region preferring high frequencies into two separate regions (hMM and hRM) possibly reflecting the homolog belt fields in the macaque (Kusmierek and Rauschecker, 2009). At the medial junction of the belt regions, located on the medial crown of HS, another reversal in frequency is found. We interpret it as reflecting regions hCM and hCL, each containing a fully tonotopic gradient (Hackett et al., 1998; Kajikawa et al., 2005), but with strong predominance of responses to high frequencies. The lateral belt (middle part of Figure 5) contains a single high-to-low tonotopic gradient in postero-medial to antero-lateral direction (that follows the tonotopic gradient in hA1). Although only one tonotopic gradient occupies this region, it may comprise two functionally separate subdivisions. The medial part of this region includes a full high-to-low tonotopic gradient, and may correspond to the human homolog of lateral belt regions ML in the monkey (Hackett et al., 1998). Conversely, the lateral part of this region is strongly tuned to low frequencies and overlaps with the speech/voice sensitive region on lateral HS/middle STG (white outlines in Figure 5; Belin et al., 2000; Moerel et al., 2012). We interpret this region as reflecting hAL. The low frequency tuning of this region could reflect a uniquely human property, deriving from the need to process the low-frequency spectral energy of voices and speech. Furthermore, based on its anatomical location (lateral to the hA1/hR boundary) and low frequency tuning, this region may correspond to the human homolog of the “pitch” region (Griffiths and Hall, 2012), which was shown to contain a large proportion of low-frequency tuned neurons responding selectively to missing fundamental pitch (Bendor and Wang, 2005).

The human parabelt may be situated posterior-laterally to the lateral belt (right part of Figure 5; Galaburda and Sanides, 1980; Hackett et al., 1998; Morosan et al., 2005). This region is substantially larger in the left than in the right hemisphere. Correspondingly, while the parabelt may be largely situated on the external part of PT in the left hemisphere, it may be shifted laterally onto posterior STG/STS in the right hemisphere. Systematic research across individuals is required to confirm this proposal. The border between lateral belt and parabelt cannot be reliably identified non-invasively. Progress in making this division may be expected from MRI explorations of myelin contrast. In addition to the cluster with densest myelination, which was interpreted as reflecting the core, De Martino et al. (2014) identified three regions with varying patterns of myelination throughout the cortical depth. If and how these clusters may be related to belt/parabelt divisions is topic of further investigations. Alternatively, we may learn more about the parabelt regions by exploring their functional properties. Additional gradients of tonotopy and tuning width occupy these regions on the STG and the posterior end of the temporal plane. Within these regions, speech/voice sensitive regions reside. Mapping the response properties of these regions (e.g., tuning to temporal/spectral modulations, latency; Santoro et al., 2014) and the transformations of sound representations may give insights in the topology of the auditory parabelt.

### Hemispheric differences in the topography of human auditory cortex

While a subset of tonotopy studies observed hemispheric biases, reporting a more prominent tonotopic organization in either left (Wessinger et al., 1997) or right hemisphere (Bilecen et al., 1998; Langers et al., 2007), in others (Woods et al., 2009; Da Costa et al., 2011; Striem-Amit et al., 2011; Moerel et al., 2012) the main tonotopic axis in the vicinity of HG is similar across hemispheres and also the orientation of the narrowly tuned region with respect to HG appears stable across hemispheres (Moerel et al., 2012). The additional frequency regions reported above (on middle STG, in the FTS, and on the posteromedial end of HG) are consistently observed in the right hemisphere as well as in the left. The only exception may be the additional frequency gradients posterior to the main high-low-high frequency gradient, extending from HS covering PT and posterior STG. While the additional low frequency reversal on posterior STG/lateral PT (region 6A in Figure 3D) is present in the right hemisphere, the right hemisphere tonotopic map may miss a part in medial PT (region 6B in Figure 3D).

We observed an increase in intersubject variability in the right hemisphere tonotopic maps compared to the left hemisphere tonotopic maps (Moerel et al., 2013). It is not clear whether this increased variability is due to poorer across-subject alignment of macroanatomy, or if it reflects true variability in the tonopic pattern. In terms of gross macroanatomy, the right supratemporal plane is shifted anteriorly and laterally compared to the left supratemporal plane (shift of approximately 7 and 5 mm anteriorly and laterally, respectively; Rademacher et al., 2001), and the STS is deeper in the right than left hemisphere (Ochiai et al., 2004). Alternatively, the SF is longer and more horizontal in the left hemisphere than the right (Steinmetz et al., 1990; Ide et al., 1996), and PT is larger in the left than the right supratemporal plane (Geschwind and Levitsky, 1968). These asymmetries in macroanatomy already exist in infants (Witelson and Pallie, 1973; Glasel et al., 2011), suggesting that they are genetically determined. Interestingly, a recent study showed that across-subject variability in macroanatomical landmarks and functional responses increases with development in the right compared to the left hemisphere. This suggests that the right supratemporal plane may be shaped by unique individual developmental experiences (Bonte et al., 2013). The increased inter-subject variability in the right hemispheric tonotopic maps compared to the left is in accordance with this suggestion. Interhemispheric differences have also been reported at microanatomical level. As PT is larger in the left hemisphere, cytoarchitectonically defined region Tpt on PT is larger in the left hemisphere as well (Galaburda and Sanides, 1980). While the size of left and right PAC is similar (Galaburda and Sanides, 1980; Rademacher et al., 2001), regions PaAi and PaAe that are located posterior to HG and anterior to Tpt are larger in the right hemisphere (Galaburda and Sanides, 1980). Therefore, we expect lateral belt regions (middle column in Figure 5) to be wider in the right hemisphere compared to our working model.

The left and right hemispheres have different functional roles in sound processing. Studies showed a relative dominance for language processing and tonal, music, and voice processing for left and right hemisphere, respectively (Zatorre, 1988; Belin et al., 2000; Hickok and Poeppel, 2000; Scott et al., 2000). Hemispheric biases in acoustic feature processing reflect the computational demands arising from this task-dependent specialization, such that the left hemisphere is relatively optimized for temporal processing (Shannon et al., 1995; Liégeois-Chauvel et al., 1999; Zatorre et al., 2002), while the right hemisphere is relatively superior in fine spectral processing (Liégeois-Chauvel et al., 2001; Zatorre and Belin, 2001; Zatorre et al., 2002). To the best of our knowledge, the relation of this functional asymmetry to the underlying tonotopic maps has not been studied. As such, it is a challenge for future research to explore how these reported hemispheric biases in spectrotemporal processing are reflected within the different auditory fields.

### Conflict of interest statement

The authors declare that the research was conducted in the absence of any commercial or financial relationships that could be construed as a potential conflict of interest.
